# A novel method to examine individual RNA structures within a mixed population reveals insights into the HIV-1 RNA dimerization process

**DOI:** 10.1186/1742-4690-10-S1-P41

**Published:** 2013-09-19

**Authors:** Julia Kenyon, Liam Prestwood, Stuart Le Grice, Andrew Lever

**Affiliations:** 1 Department of Medicine, University of Cambridge, Cambridge, UK; 2 HIV Drug Resistance Program, Center for Cancer Research, National Cancer Institute, Frederick, MD 21702-1201, USA

## 

Different models have been proposed for the HIV-1 RNA monomer and dimer structures, often by studying mutants that disrupt the palindromic nature of the dimerisation initiation site (DIS) to stabilise the monomer. We have developed a new technique known as “ingel SHAPE (selective 2’OH acylation analysed by primer extension)”. Individual RNA structures within a mixed structural population are isolated by electrophoresis under native conditions, and secondary structural probing takes place within the gel. High-throughput analysis generates a secondary structural map of each RNA, making it possible to examine the structures of different conformers under native conditions, without the need to stabilise each structure by mutagenesis or by using non-physiological buffers. We have validated the technique using TAR RNA, and show it to be accurate and highly reproducible. We have then used it to show that under native conditions the unspliced HIV-1 RNA monomer and dimer have different acylation sensitivities across the packaging signal region, but that the DIS is unreactive in both monomer and dimer. These data imply that a structural switch takes place; further examination showed that our results fit most closely to a model where, in the monomer, the dimerisation initiation site interacts with the U5 region, and that the native dimer structure corresponds closely with previously proposed structures of the monomeric RNA. This new technique allows us to visualise the nucleotides involved in the RNA structural switch, and those that remain static.

